# Sustainability Recommendations and Practices in School Feeding: A Systematic Review

**DOI:** 10.3390/foods11020176

**Published:** 2022-01-10

**Authors:** Emanuele Batistela dos Santos, Dayanne da Costa Maynard, Renata Puppin Zandonadi, António Raposo, Raquel Braz Assunção Botelho

**Affiliations:** 1 Department of Food and Nutrition, Federal University of Mato Grosso, Cuiabá 78060-900, Brazil; emanuelebatistela.ufmt@gmail.com; 2 Department of Nutrition, University of Brasília, Brasília 70910-900, Brazil; day_nut@yahoo.com.br (D.d.C.M.); renatapz@unb.br (R.P.Z.); 3 CBIOS (Research Center for Biosciences and Health Technologies), Universidade Lusófona de Humanidades e Tecnologias, Campo Grande 376, 1749-024 Lisboa, Portugal

**Keywords:** school feeding, school meals, sustainability

## Abstract

Considering the importance of schools for sustainable food offers and the formation of conscientious citizens on sustainability, this systematic review aimed to verify the recommendations on sustainability in school feeding policies and the sustainability practices adopted in schools. The research question that guided this study is “what are the recommendations on sustainability in school feeding policies and the sustainability practices adopted in schools?”. This systematic review was prepared according to PRISMA, and its checklist was registered in PROSPERO. Specific search strategies for Scopus, Web of Science, Pubmed, Lilacs, Google Scholar, and ProQuest Dissertations & Theses Global were developed. The included studies’ methodological quality was evaluated using the Meta-Analysis Statistical Assessment and Review Instrument (MASTARI). A total of 134 studies were selected for a full reading. Of these, 50 met the eligibility criteria and were included in the systematic review. Several sustainability practices were described. The most cited are school gardens and education activities for sustainability. However, actions carried out in food services were also mentioned, from the planning of menus and the purchase of raw materials (mainly local and organic foods, vegetarian/vegan menus) to the distribution of meals (reduction of organic and inorganic waste: composting, recycling, donating food, and portion sizes). Recommendations for purchasing sustainable food (organic, local, and seasonal), nutrition education focused on sustainability, and reducing food waste were frequent; this reinforces the need to stimulate managers’ view, in their most varied spheres, for the priority that should be given to this theme, so that education for sustainability is universally part of the curricula. The importance of education in enabling individuals to promote sustainable development is reaffirmed in Sustainable Development Goal 4 (SDG 4). The development of assessment instruments can help monitor the evolution of sustainable strategies at schools and the main barriers and potentialities related to their implementation.

## 1. Introduction

School feeding programs, widely spread across the globe, are recognized as an essential strategy for achieving goals in various sectors of society, including education, health, social protection, and agriculture. Recognized as the most prominent social protection network globally, even with the effects suffered by the COVID-19 pandemic, they appear as a robust investment in human capital that will guarantee the economic growth of nations [1]. The relationship between school feeding and educational and nutritional outcomes is widely investigated in the literature [2,3,4,5]. However, more recently, its role in conducting actions aimed at sustainability has been studied to mitigate the global challenges that threaten human and planet health in the 21st century [6,7,8].

A product of the concern with the environmental impacts derived from the world pattern of production and consumption in the second half of the 20th century, the term sustainable development refers to satisfying the needs of the present without compromising the ability of future generations to meet their own needs [9]. Integrating the sustainability pillars is necessary to increase productive potential, guaranteeing equal opportunities for all without putting the environment at risk [10]. The three main pillars (environmental, economic, and social) have been studied for years, and, recently, the cultural and health pillars were also linked to sustainability [11]. 

Through school meals and educational practices, students become aware of the impacts of individual and collective choices [6,12,13,14], consequently generating better environmental, economic, and social outcomes. The essential role of education for achieving a more sustainable future in environmental, economic, and social aspects was recognized by the United Nations in the Decade of Education for Sustainable Development, which aimed to integrate the values, principles, and practices of sustainable development in all aspects of education [15]. Therefore, education for sustainability is a powerful tool capable of providing the knowledge, skills, and awareness needed by young people to deal with the various problems that threaten the integrity of the planet and human health and well-being [16]. In this sense, it is essential to conduct a process that considers, in addition to global issues, those that are local and common to the participants’ routine and that integrates a holistic perspective, allowing for informed decision-making, individually or collectively [15,17]. The literature also highlights the importance of using school meals as a tool for nutritional education and education for sustainable consumption and practical learning activities, such as school gardens, cooking activities, and field visits to small local farmers [6,12,18,19,20].

The relationship between sustainability and school feeding also occurs at the level of decisions made at all stages of meal production. It is known that food production is associated with significant environmental impacts. Although elements before or after the meals’ preparation are responsible for most of these effects (such as field production, transport, and food waste) [21,22,23], the choices made by school food services influence this dynamic, determining the degree to which they employ actions to mitigate the environmental impacts generated in this process. Some examples are the purchase of organic and local food, encouraging the consumption of fresh vegetables at meals, controlling the supply of meat, and actions such as adjusting portions, donating food, composting, purchasing products with minimum packaging, recycling, and reducing energy and water consumption [24,25,26,27,28,29,30].

The adoption of sustainable practices often generates results that simultaneously reach the different dimensions of sustainability. Reducing energy and water consumption in the production of meals and adjusting the size of food portions, for example, can represent actions of economic and environmental sustainability [29]. Also, the donation of food, raw or prepared, provided that it is in perfect hygienic and sanitary condition, can be observed from social and environmental sustainability perspectives [26,27].

Establishing a close relationship between school meals and small farmers to purchase locally sourced food favors the increase in income and class organization. Consequently, the economic development of the region, as well as contributing to the food and nutritional security of farmers and their families [31,32], goes beyond environmental to social and economic sustainability dimensions. Due to the benefits for both students and farmers widely recognized in the literature, the practice of buying local food is encouraged by public policies for school meals in different parts of the world [33,34].

Therefore, the range of activities involved with school feeding generates unique challenges and opportunities from the point of view of sustainability in the environmental, economic, and social dimensions [7,8,12,35]. In this sense, school feeding programs are part of the strategies used to achieve the Sustainable Development Goals of the 2030 Agenda [36]. 

Although school feeding policies in some countries already present recommendations on sustainability in their guiding principles [34,37,38], and the literature presents different sustainability practices employed in this context, bringing to light a body of evidence on this topic will be helpful for decision-makers at the government level to create or even revise guidelines for their school feeding policies, incorporating the principles of sustainability. Therefore, the research question that guided this study is “What are the recommendations on sustainability in school feeding policies and the sustainability practices adopted in schools?”. In addition, the findings may help policymakers and members of the school community, within their local context, in the development of sustainability practices linked to school and school feeding. Considering the importance of schools to offer sustainable food and in the formation of conscientious citizens who are able to act on the challenges related to sustainability in the contemporary world [6], this systematic review aimed to verify the recommendations on sustainability in school feeding policies and the sustainability practices adopted in schools.

## 2. Materials and Methods

This systematic review was prepared according to the Preferred Reporting Items for Systematic Reviews and Meta-Analyses (PRISMA), and its checklist [39] was registered in PROSPERO [CRD42021264978]. The protocol was performed according to the following steps.

### 2.1. Inclusion and Exclusion Criteria

The inclusion criteria were studies that described the recommendations on sustainability in school feeding policies and the sustainability practices adopted in schools, in environmental, social, and/or economic aspects, with no date and language limits. Legislations of school feeding policies and programs found in the studies reference lists had their full text analyzed to identify sustainability recommendations. The exclusion criteria were: (1) comments, letters, conferences, reviews, abstracts, reports, undergraduate works, discussion papers, and books, (2) studies carried out outside schools or in which the school was not responsible for the action, (3) studies in which practices were not performed or studies where activities were punctual, (4) studies focused on the supplier or that only reported purchases, and (5) studies that did not describe sustainability practices (Appendix A). 

### 2.2. Information Source

Detailed individual search strategies were developed for each database: MEDLINE via Pubmed, Embase, Scopus, Web of Science, and Lilacs. A search for gray literature was performed on Google Scholar and for dissertations and theses in ProQuest Global. Additionally, we examined the reference lists of the selected articles as relevant studies could have been missed during the data search. The last search in all databases was carried out on 30 June 2021.

### 2.3. Search Strategy

The appropriate combinations of truncation and keywords were selected and adapted for the search in each mentioned database (Table S1—Supplementary Materials). We used Rayyan software (Qatar Computer Research Institute (QCRI)) to select and exclude duplicate articles, and all references were managed by Mendeley desktop software.

### 2.4. Study Selection

Two phases were necessary for the selection. In phase 1, researchers I (EBS) and II (DCM) independently reviewed the titles and abstracts of all references identified from databases. EBS and DCM excluded the articles that did not meet the eligibility criteria. In phase 2, the full texts of the selected articles were fully read by the same reviewers (EBS, DCM), and only those that met the inclusion criteria were included. In both phases, the disagreements were discussed until a consensus was reached between the two reviewers. A third reviewer (RBAB) made the final decision in situations without consensus. EBS, an examiner, critically evaluated the list of references of the selected studies. Additional studies were added by the third examiner (RBAB) and the expert (RPZ).

### 2.5. Data Collection Process

Two reviewers independently (EBS, DCM) collected the following characteristics from the selected studies by authors and year of publication, country of research, the objective of the study, methods, sustainability dimensions, and main results referring to the identified sustainability practices. Calibration exercises were conducted before starting the review to ensure consistency among reviewers. Disagreements were solved by discussion, and the third reviewer (RBAB) adjudicated unresolved disagreements. These data were synthesized by three reviewers (EBS, DCM, and RBAB) using a standardized table containing the following information: reference, authors, year, country, objectives, type of school management (public, private), teaching stage (according to the teaching stages of each country), participants (individuals, schools, or municipalities), sustainability dimensions (environmental, economic, and social), and main results referring to the identified sustainability practices.

### 2.6. Risk of Individual Bias in the Included Studies

The quality criteria were synthesized using a statistical review assessment instrument (MASTARI) and the Joanna Briggs Institute protocol to assess the risk of bias in the studies. The instrument for assessing the risk of bias included seven questions:

1. Were the practices identified characterized?
2. Has the practice been implemented in schools?
3. Did the practices present a positive implementation response?
4. Was the study design appropriate?
5. Was the statistical analysis adequate to the objective of the study?
6. Did the results answer the main question?
7. In the case of the schools, was the sample of establishments selected for analysis representative and randomly determined?

The categorization of the risk of bias followed the percentage of “yes” score: “High” for up to 49%, “Moderate” for between 50 and 69%, and “Low” for more than 70%.

## 3. Results

**Figure 1:**
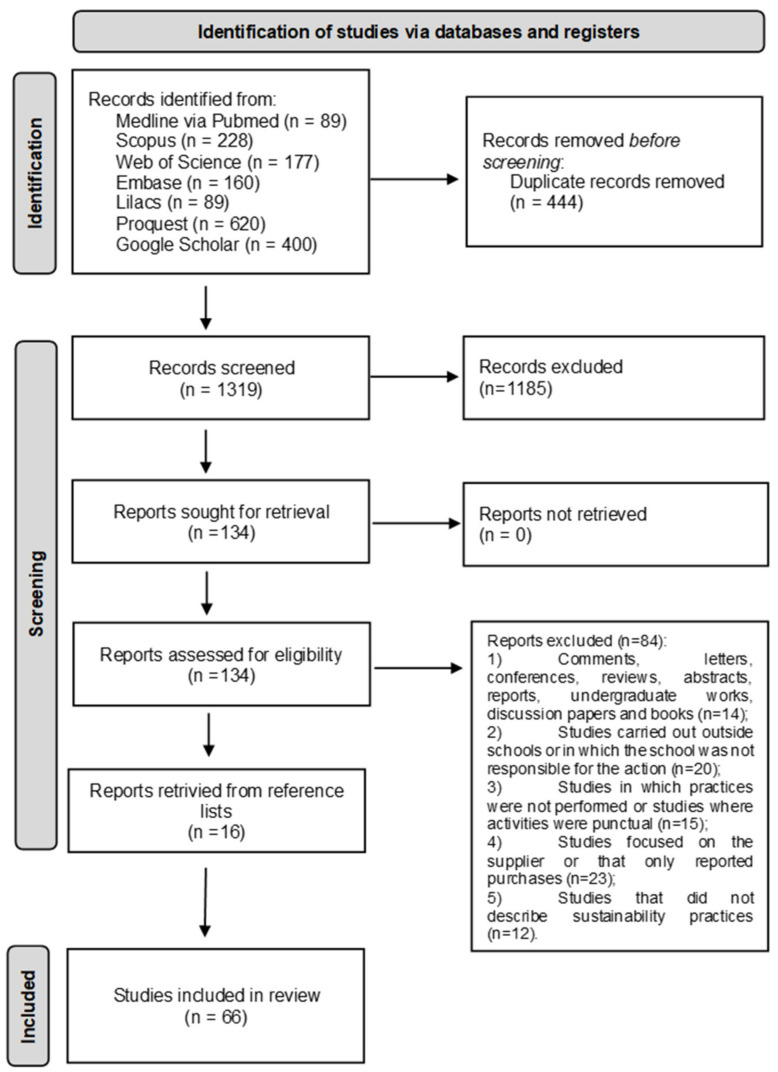
Flowchart of the systematic review search process. Adapted from PRISMA protocol.

The researchers retrieved 1763 studies from the electronic databases; 1319 titles and abstracts were evaluated after removing the duplicates, and, after reading the abstracts, 134 studies were selected for a full reading. Of these, 50 met the eligibility criteria and were included in the systematic review. At the same time, recommendations on sustainability were found in 11 governmental school feeding policies or programs and 5 in other available non-governmental school feeding programs/initiatives retrieved from the studies’ reference lists (Figure 1). The latest available versions were evaluated for governmental and non-governmental school feeding policies or programs.

### 3.1. Studies Characteristics

Regarding sustainability practices, the studies included (*n* = 50) were conducted between 1991 and 2021, in the following countries: United States (*n* = 22), Brazil (*n* = 7), Spain (*n* = 3), Italy (*n* = 3), South Africa (*n* = 2), Canada (*n* = 2), England (*n* = 2), Denmark (*n* = 1), Finland (*n* = 1), Ghana (*n* = 1), India (*n* = 1), Japan (*n* = 1), Wales (*n* = 1), Kenya (*n* = 1), and Tanzania (*n* = 1). A parallel study was carried out in the United States and Cuba. The characteristics of the analyzed studies are presented in Table 1.

In the United States, where the largest number of studies was identified (*n* = 22), sustainability practices mainly involved educational activities for sustainability [20,44,53,56,57,59,60,61,69], waste reduction [40,41,42,46,49,61,76], school gardens [20,46,55,59,60,61,64], and participation in programs that promote closer ties between schools and producers [46,48,69,70,73,77]. Practices such as food donation [49,64,69,76], strategies for the rational use of water and energy [42,61], the offer of vegetarian/vegan menus or with reduced meat supply [25,46], and the use of local and organic foods (not mentioning participation in specific programs for this purpose) [59] were less mentioned. The study was carried out in parallel in the United States and Cuba in the context related to the experiences of urban school gardens in Philadelphia (USA) and Havana (Cuba) [51].

In Canada, the identified sustainability practices (*n* = 2) involved school gardens, purchase of local and organic foods, participation in programs that promote closer ties between schools and local farmers, waste reduction, and the use of vegetarian dishes [24,71]. In Brazil, the only country in Latin America in which studies were identified (*n* = 7), the most cited practices were related to school gardens [14,45,74,75,79] and educational activities for sustainability, including training for those involved in the operationalization of the National School Feeding Program (PNAE) [14,68,74,79]. However, the studies also cited activities to reduce waste [14,45,75], use of local [14] and regional [62] foods, partnerships for the development of sustainability activities (such as environmental preservation), and to encourage both the use of organic food and to value the work of those involved in all food production [68]. 

Among European countries, three studies were conducted in Spain and Italy, two in England, and one in Denmark, Finland, and Wales. The most common practices involved were buying local and organic food [8,30,47,58,67]. In Italy, socio-environmental criteria in hiring school food services were also mentioned [47]. Among the European studies, practices linked to changes in menus or portions were also identified [8,26,27,52], sustainability certification [26], waste reduction and energy and/or water savings [26,50], participation in a program to encourage sustainable eating [12,54], school garden [50], and practices aimed at social sustainability (such as food donation and the use of food from land confiscated from criminal organizations) [8,26,27].

On the African continent, two studies were identified in South Africa [43,66] and one in Ghana [63], Tanzania [78], and Kenya [65]. The identified practices focused on activities related to school gardens [43,66,78]. However, the teaching of agroforestry practices, water-saving, and the development and adoption of a meal planning package nutritionally balanced meals, with locally sourced ingredients, were also identified in the studies [43,63,65]. 

Among Asian countries, one study was identified in Japan [35] and one in India [72]. In Japan [35], the study demonstrated that the reinforcement of social norms not to waste and factors related to the planning of menus, pedagogical practices, and recycling and composting activities, contributed to reducing the food waste in schools. In India, the sustainable management of supply chains was studied based on one of the companies responsible for the school feeding program, which considers the integration of economic and non-economic issues in the generation of value in the supply chain [72].

In 20% of the studies (*n* = 10), it was impossible to identify information about the responsibility for managing schools. Most studies were performed in public schools (64%; *n* = 32). Studies conducted in public and private schools corresponded to 14% (*n* = 7), and only one study was conducted in a private school. All stages of the education system, including the earliest (pre-school) and the final years (high school), were mentioned, but the stage referring to primary or elementary education was identified in most studies (68%; *n* = 34). In 27.4% of the studies (*n* = 14), it was impossible to obtain this information. The studies used quantitative (30%; *n* = 15), qualitative (48%; *n* = 24), or mixed methods (22%; *n* = 11).

### 3.2. Identified Sustainability Practices

**Figure 2:**
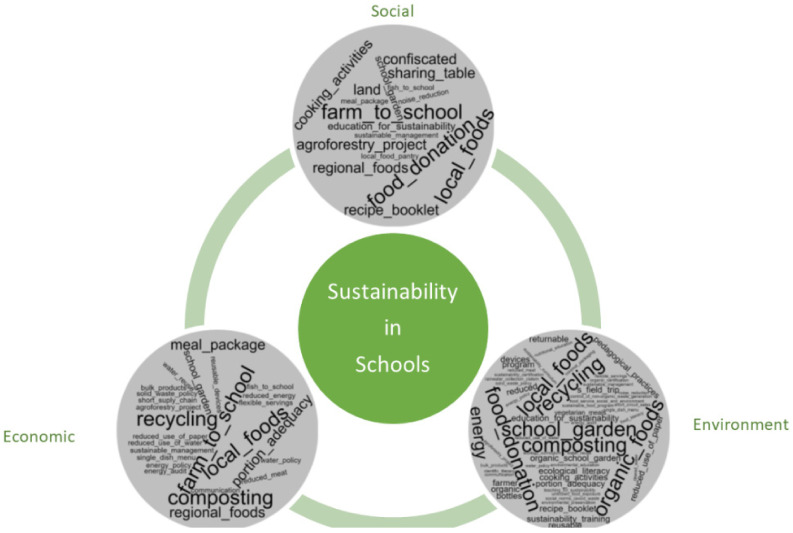
Identified sustainable practices in schools according to the environmental, economic, and social dimensions.

The environmental dimension of sustainability was identified in all studies that cited sustainability practices (*n* = 50), alone (26%; *n* = 13), or together with the other considered dimensions. In most studies (44%; *n* = 22), it was possible to identify practices related to the three sustainability dimensions (environmental, economic, and social). The combination of environmental and social dimensions was identified in 14% of the studies (*n* = 7), and the environment with economic dimension in 16% (*n* = 8). It is important to highlight that for identifying the sustainability dimensions, some practices, in isolation, represented the attendance of more than one of the mentioned dimensions. In contrast, in other cases, the identification of different sustainability dimensions in the same study resulted from different practices cited by the authors. Sustainability practices adopted in schools are presented in Table 1. The activities were described according to the dimensions of sustainability (Figure 2).

The involvement with school gardens and education activities for sustainability represented the most commonly reported practices, being identified in 36% (*n* = 18) [14,20,24,43,45,46,50,51,55,59,60,61,64,66,74,75,78,79] and 28% (*n* = 14) [14,20,35,44,51,53,56,57,59,60,61,69,74,79] of the studies, respectively. Among the studies that described the use of school gardens, 8% (*n* = 4) [14,45,61,75] described the cultivation of organic foods and, in the context of education practices for sustainability, travel field studies [59,60] and cooking activities [14,20,44,69] were also cited. 

Another frequently cited category of sustainability practices concerns schools food supply initiatives. Actions to purchase or encourage the employment of local or short-chain foods, including participation in programs such as “Farm to School” and “Fish to School” were cited by 26% (*n* = 13) of the studies [8,14,24,30,46,48,59,67,69,70,71,73,77] and organic foods were observed in 18% (*n* = 9) [8,24,30,45,47,58,59,67,68]. Participation in a sustainable food consumption promotion program (4%; *n* = 2) was also identified [12,54]. Although little mentioned, practices that integrated socio-environmental and economic dimensions in the contracts (4%; *n* = 2), the use of regional (2%; *n* = 1), seasonal (2%; *n* = 1), and produced foods in lands confiscated from criminal organizations, were also observed [8,30,47,62,72].

Regarding the adoption of measures to reduce waste, recycling [35,40,41,42,46,49,50] and composting [24,26,35,46,49,61,76] were the most reported, described in 14% (*n* = 7) of the analyzed studies. These represent important strategies for the control of organic and inorganic waste. Food donation was a practice identified in 10% (*n* = 5) of the studies [26,27,49,64,76] and food portion size adjustment in 6% (*n* = 3) [26,27,35]. Sharing tables [76], single-course menu [8], reinforcement of social norms [35], donation of food waste for animal feeding [49], team communication to adjust the amount produced, and noise reduction in the cafeteria to allow a more comfortable environment [26] were also identified practices for the reduction of organic waste, to a lesser degree than the others previously mentioned. Other less cited practices involving the control of non-organic waste generation were the use of reusable devices [40,45], the purchase of products in bulk or with minimum packaging [24,40], the use of returnable bottles [14], reduced use of paper [26], or the replacement of mineral water by filtered water [8]. 

The adoption of strategies that involved saving water or energy was cited in 10% (*n* = 5) of the studies [26,42,43,50,61], represented by activities such as reduced use of energy, energy audits, reduced use of water, and installing a cistern for collection rainwater or irrigation system for water reuse.

As for menu actions, practical studies were identified as offering vegetarian/vegan meals [24,46,52], reducing meat supply [25], planning a menu that included the exposure of students to unfamiliar or unappreciated foods [35], and the adoption of a meal planning package that facilitated the planning of nutritionally balanced meals with locally sourced ingredients [63]. 

Although mentioned in only one study, partnerships for the development of sustainability activities (such as environmental preservation) and certification were also identified [26,68].

### 3.3. Sustainability Recommendations in School Feeding Policies/Programs

Among the 11 policies under government responsibility identified in the studies that mentioned sustainability recommendations, 73% (*n* = 8) were national in scope, 18% (*n* = 2) state, and 9% (*n* = 1) municipal. The European continent had the highest number of policies/programs (64%; *n* = 7), identified in Italy, England, Finland, Spain, Sweden, and Germany [37,80,81,82,83,84,85]. School feeding policies/programs were also identified in Brazil, Japan, and the United States [34,86,87,88,89] (Table 2).

The most mentioned aspects were the origin and type of food used in school meals, such as organic, local or shorter transport distances, seasonal, agroecological, and sustainable [34,37,80,81,82,83,84,85,86,87]. Other examples cited in this category, although less frequently, were reducing meat, increasing consumption of vegetables, reducing carbon emissions, typical foods, and respecting local traditions [34,37,82,84,86].

Nutrition education practices and sustainable consumption and recommendations for reducing food waste and non-organic residues were also identified [34,37,80,81,84,86,88,89]. School gardens, fair trade, valuing workers involved in food production, energy reduction, and animal welfare, although observed, were less frequent [34,37,80,84,86,88].

Regarding other non-governmental programs and initiatives (*n* = 5), two were identified in the United States [90,91], two were global in scope [92,93], and one was identified in England [94]. The set of activities observed in these programs involved encouraging the purchase of local, seasonal, and sustainable food [91,92,94], school gardens [91,94], visits to local farmers [91,94], cooking and nutritional education activities [91,94], waste reduction [90], and specific actions for each school to train people to generate environmental and sustainability awareness [93] (Table 3).

### 3.4. Risk of Bias

Among the studies analyzed, 49 had a low risk of bias and 1 had a moderate risk. All studies implemented the practices and answered the main research question (Table S2—Supplementary Materials).

## 4. Discussion

In 2019, the EAT-The Lancet Commission established universal strategies and recommendations to achieve food system transformation, striving for human health and environmental sustainability. The need to improve availability and access to healthy foods from sustainable food systems and educate individuals on these topics using food programs was reinforced [95]. Therefore, the school is an opportune locus for sustainability practices. The environmental, economic, and social effects of the actions carried out in school food services and the education process will echo both in society’s present and future.

Vegetable gardens and education activities for sustainability were the most cited practices among the studies. School gardens are essential tools to support community and school feeding programs by using their produce in student meals and training vegetable growing skills [19,43,66]. However, the support of the school administration, the availability of space and resources to purchase tools and supplies, teacher training, the integration of the garden into the school curriculum, sharing activities with community members, and the presence of a coordinator to organize activities are identified as key factors in determining the results of the implementation and continuity of school gardens [66,96,97]. 

Education plays a central role in enabling students to think and act critically on current and future global challenges, including climate change, environmental degradation, biodiversity loss, poverty, and inequality [16]. Therefore, the literature has strongly recommended and evidenced the association between school gardens and educational processes aimed at health, environment, and sustainability [19,24,61]. Gonsalves et al. [19] emphasize the role of school gardens “in environmental and nature education, in local food biodiversity and conservation, food, eco-literacy, diets, nutrition and health, and agricultural education, contributing to the search for a food system more sustainable.”

The importance of education in enabling individuals to promote sustainable development is reaffirmed in Sustainable Development Goal 4 (SDG 4). SDG 4 is integrated with other indivisible goals, comprising the environmental, economic, and social dimensions of Agenda 2030, an action plan for people, the planet, and prosperity [98]. 

In line with this purpose, schools can observe efforts to integrate environmental education, ecological literacy, or education for sustainability in their curricula [14,56,57,60,69]. However, a recent study [99] that assessed the extent to which environmental issues are integrated into primary and secondary education policies and curricula in 46 member states of the United Nations Educational, Scientific, and Cultural Organization (UNESCO) demonstrated that despite 92% of the documents making some reference to environmental issues, the depth of this inclusion was low. Terms such as “climate change” and “biodiversity” were rarely mentioned. What has been done is not enough to ensure that learning helps in confronting current global challenges. The document recommends, among other actions, that greater emphasis needs to be placed on environmental issues in education, integrating them into curricula and overcoming the focus on cognitive knowledge, and training all teachers and school leaders in education for sustainable development [99]. Also, considering the food system’s environmental, economic, and social impacts, the efforts to integrate themes involving sustainable food consumption into educational practices are worthy of note [12,44]. These approaches are fundamental because education can increase adherence to different sustainability practices since, although observed in this study, many had a low frequency of realization.

The use of local or organic foods, often set by government regulations, stems from concerns about students’ health, the living conditions of farmers, and the environment [34,47,48,70,100]. Initiatives involving the use of local and organic foods were also frequently cited among the analyzed studies [8,14,24,30,47,58,59,67].

The creative food purchase policy incorporates social and environmental criteria into the contracts, going beyond economic considerations and encouraging the purchase of local food [33]. Examples of buying local and organic food are worldwide, as in the Brazilian case at the National School Feeding Program, where the purchase of food from local farmers is a compulsory item provided in its legal framework. Organic food is preferred in public purchases, the standardized instruments for this type of purchase in the country’s program [34]. Through Home Grown School Feeding, the World Food Program (WFP) works with governments in 46 countries to develop national policies that provide adequate food for students and ensure local development by purchasing food from family farmers [92]. There are also experiences linked to the Farm To School programs, which purchase local food and develop educational activities related to agriculture, food, health, and nutrition [70,71]. All initiatives that connect schools to family farmers are vital because the benefits of school meals go beyond the boundaries of schools and reach family farmers. These involve economic (increase in income, price support, and inclusion in the market), social (food security, living conditions, and social inclusion), and environmental (crop diversification and greater production of organic food) aspects [31]. 

Reducing food waste and controlling non-organic waste represent initiatives that must be implemented in school food services. Studies in different parts of the world have demonstrated that these places are major food waste generators, causing environmental, economic, and social impacts [101,102,103,104]. Concerning non-organic waste, a study carried out in northern Colorado, USA identified that factors such as the speed of the service line, the quality of food, the cost, and the difficulty managers have in understanding the impact of their decisions at a systemic level, affected the ability to reduce or recover these wastes [105].

In our systematic review, composting was the most cited practice for reducing the generation of organic waste and recycling non-organic waste. However, it is important to highlight that even among the studies in which the performance of waste management practices was cited, they were often not reported among the participants or were reported by a minority of them [26,40,41,42,46,49,76], demonstrating that adopting waste reduction strategies in school meals is not yet routine practice. 

Among the strategies to promote the reduction of food waste, the literature discusses the importance of integrating this theme and the sustainability of the food system in pedagogical practices, in addition to actions aimed at improving operations and planning, team communication, and the involvement of students in waste management activities [35,102]. Food donation can represent a successful experience to mitigate the impacts of the production of meals by reducing waste and serving people in vulnerable situations, with relatively few investments [27]. However, the main barriers related to food donation and food recovery in this context involve concerns about responsibility, cost, inconsistent food waste, policy confusion, and the sanitary quality of food [76]. As for non-organic waste, among other recommendations, a study indicated that school food services could incorporate packaging waste in purchasing processes, as they do not always control the packaging used by manufacturers [105].

The adoption of some strategies related to saving water and energy was mentioned among the analyzed studies. In school feeding, studies that reported the environmental impacts of the choices made by food services regarding the origin and types of food purchased (fresh or not, and from different groups, such as meat and vegetables) demonstrated a significant contribution from phases before the production of meals [21,23,106]. However, considering that during the production of meals both water and energy are essential factors for the operation of the service, the training of a school’s employees and the monitoring of the intended use of these resources is necessary. Instruments created to evaluate sustainability practices in food services, which include among their analysis categories the rational use of energy and water, are helpful tools in this regard [107,108]. 

Environmental and health damage to the population generated by how the food system has been operated is already well established [109]. Two of the factors contributing to the harmful effects of this modus operandi are meat production, especially red meat, and food waste, which are responsible, among other factors, for a significant emission of greenhouse gases into the environment and/or consumption of freshwater [23,106,110,111]. Therefore, some practices related to the offer of vegetarian/vegan menus, with a reduction in the meat offering, the adequacy of the portion sizes, or the adoption of the single-course scholar menu have been reported in the literature [8,25,26,27,35,112]. Some instruments have been proposed to allow the planning of more environmentally sustainable menus based on reducing carbon and/or water footprints while addressing nutritional, economic, and cultural dimensions [7,113,114,115,116]. In addition, the definition of criteria for planning sustainable menus in the context of school meals has also been described [117].

Other less mentioned strategies involved using regional foods, environmental certification, and the development of partnerships to carry out environmental preservation activities. 

According to Morgan [118], “the creation of a sustainable school foodservice is the litmus test of a country’s commitment to sustainable development, as it involves nothing less than the health and well-being of young people and vulnerable people”. In this sense, several efforts were made to strengthen the role of school feeding in achieving nutritionally adequate diets for students and meet the principles of sustainability in the three dimensions: environmental, economic, and social. However, it is noteworthy that, despite the general premise established in the literature of the potential effects of sustainability practices in school in mitigating global challenges, the wide scope of school feeding and the variability of characteristics and challenges experienced between different regions of the globe, including different regulations, economic, social, political and cultural conditions, demand specific solutions, adapted to each local context.

## 5. Limitations

This review has some limitations. First, it was not possible to state that the school feeding policies/programs that mentioned concerns about sustainability were exhausted, since the policies were found in the studies reference lists that had their full text analyzed. In addition, some of these policies, written in a non-English language, were translated through a translation platform. Therefore, some information may have been lost due to language barriers. Despite these limitations, these findings evidenced different recommendations that reinforced the importance of actions, which ranged from the choice of sustainable foods to the strengths of nutrition and sustainable consumption practices education.

## 6. Conclusions

There is an imminent need to ensure the prosperity of nations, anchored in the priorities of protecting the health of people and the planet and guaranteeing adequate living conditions, reducing social inequalities. It involves offering food in terms of education, enabling students to make conscious choices consistent with this need. In this sense, schools and school feeding programs have all the necessary characteristics for developing practices that aim at sustainability in the environmental, economic, and social dimensions, given their scope and the different perspectives that can be worked.

The present study identified sustainability recommendations in 16 governmental and non-governmental policies/programs. Recommendations for purchasing sustainable food (organic, local, and seasonal), nutrition education focused on sustainability, and reducing food waste were frequent.

Several sustainability practices were described in this systematic review, such as the use of school gardens and education activities for sustainability. Actions carried out in food services were also mentioned, from the planning of menus and the purchase of raw materials (mainly local and organic foods, vegetarian/vegan menus) to the distribution of meals (especially practices to reduce waste organics and inorganics such as composting, recycling, donating food, and adjusting portion sizes).

The findings reinforce the need to stimulate managers’ views, in their most varied spheres of power, for the priority that should be given to this theme, so that education for sustainability is universally part of the curricula, and so that food services can equip themselves with the knowledge and tools necessary to carry out sustainability practices in their daily activities.

Lastly, further investigations to evaluate these practices are needed to examine the evolution of their adoption and the main barriers and potentialities related to their implementation. With a specific look at the school field, assessment instruments can help with this monitoring.

## Figures and Tables

**Table 1:** Main descriptive characteristics and results from the included studies.

Author (Year) Country	Objectives	School Management (SM) Teaching Stage (TS) Participants (P) Sustainability Dimension (SD)	Main Sustainability Practices Identified
Mann (1991) [40] USA	To assess the solid waste management practices in school food, and to develop and assess a decision model for solid waste management in school food services.	SM: Public, private TS: Not informed P: School food service directors (*n* = 458) SD: Environmental, economic	Recycling, purchase of bulk products, and reusable dispensing devices.
Ghiselli (1993) [41] USA	To analyze waste and disposal practices in Indiana’s school food service, and the feasibility of reducing it through permanent service and product recycling.	SM: Public, private TS: Elementary, middle, high school P: School food service directors (*n* = 237) SD: Environmental, economic	Recycling.
Hackes; Shanklin (1999) [42] USA	To identify resource allocation decisions, policies, and procedures used by school food service directors that were based on pollution prevention, product stewardship, and sustainable development.	SM: Not informed TS: Not informed P: School food service directors (*n* = 168) SD: Environmental, economic	Recycling; energy policy: solid waste and water.
Albertse, Mancusi-Materi (2000) [43] South Africa	To illustrate how the initiation of school children into innovative technologies has fostered mechanisms of social mobilization towards enhanced food security in South Africa.	SM: Not informed TS: Not informed P: Students, parents (*n* = not informed) SD: Environmental, economic, social	Irrigation system for water reuse and school garden.
Wadsworth (2002) [44] USA	To conduct a curriculum assessment of an after-school program on food choices that minimize energy, natural resources used, and pollution generated in food processing, packaging, and transportation.	SM: Public TS: Elementary P: Students (*n* = 240) SD: Environmental	Nutritional education focused on the sustainability of the food system; Cooking activities.
Lima (2006) [45] Brazil	To analyze the management of a School food service unit in the State of Santa Catarina, based on the introduction of organic foods.	SM: Public TS: Elementary P: Representatives of the Department of Education, a state school, the School Feeding Council, students (*n* = 21) SD: Environmental	Organic school garden and feeding program; Control of non-organic waste generation.
Vogt (2006) [46] USA	To identify district/community characteristics supporting buying food locally, the perceived benefits and barriers in buying locally, and generate solutions to encountered issues in California.	SM: Public TS: Not informed P: School food service directors, farmers (*n* = 37) SD: Environmental, economic, social	Participation in the “Farm-to-School” program (local foods), school garden, recycling, composting, and vegetarian/vegan meals.
Sonnino (2009) [47] Italy	To examine how city authorities have integrated different (and at times contrasting) quality conventions in school meals in Rome.	SM: Public TS: Not informed P: Representatives of the sectors involved in school feeding (*n* = not informed) SD: Environmental, economic, social	Purchase of organic food; Adoption of social and environmental criteria for contracting food services.
Izumi, Alaimo, Hamm (2010) [48] USA	To identify why farmers, school food service professionals, and food distributors participate in farm-to-school programs and the opportunities and challenges for purchasing food at local schools.	SM: Public TS: Not informed P: School food service professionals, farmers, food distributors (*n* = 18) SD: Local, economic, social	Participation in the “Farm-to-School” (local food).
Baca (2011) [49] USA	To investigate the status of food waste management programs, recycling of packaging waste, and cost of waste hauling in school nutrition programs in the USA.	SM: Not informed TS: Not informed P: Child nutrition directors (*n* = 79) SD: Environmental, economic, social	Food donation, composting, donation of waste for animal feed, recycling.
Bennell (2012) [50] Wales	To explore the development of the Education for Sustainable Development and Global Citizenship through case studies of Welsh primary schools.	SM: Not informed TS: Elementary, middle P: Students, teachers, support staff (*n* = 46) SD: Environmental, economic	School garden, recycling, energy audit, and sustainability aspects.
Bucher (2012) [51] USA/Cuba	To examine how pedagogies of sustainability are embedded in socio-cultural contexts and policy structures and driven by the localized actions of teachers.	SM: Public TS: Elementary, primary, high school P: Teachers, community members (*n* = 12) SD: Environmental, social	Environmental education; school garden.
Jones et al. (2012) [12] England	To examine the associations between the promotion of sustainable food and student self-reported fruit and vegetable consumption and associated behaviors.	SM: Not informed TS: Elementary P: Students (*n* = 1435) SD: Environmental	Participation in a sustainable food program (education for sustainability, use of sustainable food).
Lombardini, Lankoski (2013) [52] Finland	To examine the effects of forced restriction of food choice through a natural field experiment, the Helsinki vegetarian day.	SM: Not informed TS: Elementary, high school, vocational P: Schools (*n* = 43) SD: Environmental	Vegetarian day.
O’Brien (2013) [53] USA	To explore efforts by some independent schools to develop education and act in ways that promote environmental sustainability and social equity.	SM: *Independent TS: High school P: Schools (*n* = 5) SD: Environmental, social	Education for sustainability.
Orme et al. (2013) [54] England	To report on an evaluation of the Food for Life Partnership program, a multi-level initiative in England promoting healthier nutrition and food sustainability awareness for students and their families.	SM: Not informed TS: Elementary P: Teachers, students (*n* = 152) SD: Environmental	Formation of a leadership group in a sustainable food program (tasting new dinners; visits to local farmers to buy and prepare food to share with the school).
Rilla (2013) [55] USA	To examine the design features of schoolyard gardens in the Unified School District of Los Angeles and see how they are a way to encourage community involvement.	SM: Public TS: Elementary, middleP: Schools (*n* = 5) SD: Environmental	School garden.
Shuttleworth (2013) [56] USA	To investigate the curricular, pedagogical, and assessment strategies of three teachers when they teach the social issues of sustainability education.	SM: Public, private TS: Elementary, middle, high school P: Teachers (*n* = 4) SD: Environmental, locial	Education for sustainability.
Barnett (2014) [57] USA	To examine the founding and first ten years of operation of a charter school committed to ecological literacy and sustainability.	SM: Public TS: Elementary, middle P: Founders, alumni (*n* = not informed) SD: Environmental	Ecological literacy.
Galli et al. (2014) [8] Italy	To explore the role of new public-private partnerships for promoting more sustainable school meal services, by drawing on the theory of co-production.	SM: Public TS: Not informed P: Representatives from the food service and education sector, parents (*n* = not informed) SD: Environmental, social	Short supply chain; organic food; use of food produced on confiscated land; exchange of mineral water for filtered water; single dish menu.
He, Mikkelsen (2014) [58] Denmark	To examine the possible influence of organic food policies on Danish school feeding systems on the development of healthier school food environments.	SM: Public TS: Elementary P: School food service supervisors (*n* = 92) SD: Environmental	Organic food.
Keller (2014) [59] USA	To examine how educators are fostering sustainability through cultivating nature awareness in young children.	SM: Public, independent TS: Elementary P: Scholar, education director, teachers, principal (*n* = 6) SD: Environmental, economic, social	Ecological literacy; school garden; field trips to farmers; local and organic foods.
Bamford (2015) [60] USA	To discover the relationships between educational experience and sustainability attitudes and behaviors, the motivation behind these behaviors, and establish their role in educational programs.	SM: Not informed TS: Elementary P: Students, teachers (*n* = 102) SD: Environmental, social	Sustainability curriculum; school garden; field trips.
Black et al. (2015) [24] Canada	To describe the development of a tool to assess the integration of healthy and environmentally sustainable food initiatives in schools and characterize a sample of schools using this tool.	SM: Public TS: Elementary, secondary P: Food service worker, teachers, school administrators (*n* = not informed) SD: Environmental, economic, social	School garden; composting; local, organic food with minimal packaging; vegetarian dishes.
Coe (2015) [61] USA	To understand how a school gardening program and its ecology curriculum influences students’ environmental perceptions and attitudes.	SM: Public TS: Elementary P: Students (*n* = 21), parents (*n* = 3), staff (*n* = 3) SD: Environmental	Ecology curriculum; organic school garden; rainwater collection cistern; composting.
Fabri et al. (2015) [62] Brazil	To identify and analyze the use of regional foods in the school meals of a Brazilian city.	SM: Public TS: Not informed P: City (*n* = 1) SD: Environmental, economic, social	Regional food.
Strohl (2015) [20] USA	To investigate how science education is structured to develop scientifically literate students.	SM: Not informed TS: Elementary P: Teachers (*n* = 2) SD: Environmental	School garden; food literacy; scientific literacy; cooking activities.
Triches (2015) [14] Brazil	Report the actions taken with schoolchildren in a municipality, combining changes in food consumption and production and linking health and sustainability.	SM: Public TS: Not informed P: City (*n* = 1) SD: Environmental, economic, social	Local foods; organic school garden; teaching for sustainability; cooking activities; use of returnable juice bottles.
Fernandes et al. (2016) [63] Ghana	To describe the adaptation of the School Meals Planner Package to reality in Ghana during the 2014 to 2015 school year.	SM: Public TS: Not informed P: Districts of Ghana (*n* = 42) SD: Environmental, economic, social	Meal package plan (local food).
Bareng-Antolin (2017) [64] USA	To identify practices, perceived benefits, barriers, and resources needed to implement and maintain a gardening program in high schools.	SM: Public; Private TS: High school P: Teachers; School administrators or staff, community volunteers, support organizations (*n* = 42) SD: Environmental, economic, social	School garden; food donation.
Borish, King, Dewey (2017) [65] Kenya	To understand how a school feeding and agroforestry program impacts the surrounding community’s human, financial, natural, and social capital.	SM: Public TS: Elementary P: Community members (*n* = 64) SD: Environmental, economic, social	Agroforestry project (teaching on agroforestry practices).
Laurie, Faber, Maduna (2017) [66] South Africa	To evaluate knowledge, perceptions, and practices about food production among students and educators, management, and gardening activities in the National School Feeding Program schools.	SM: Public TS: Elementary P: Garden administrators, garden workers, teachers, students (*n* = 3355) SD: Environmental, economic	School garden.
Soares et al. (2017) [67] Spain	To identify and characterize initiatives that promote the purchase of locally-sourced foods to supply schools and the schools carrying out the initiatives.	SM: Public, private TS: Kindergarten, elementary, high school, special school P: Informants from the Ministries of Education and Agriculture (*n* = Not informed) SD: Environmental, economic, social	Local foods; organic food.
Garcia (2018) [68] Brazil	To analyze the actions of the National School Feeding Program in the city of Marechal Cândido Rondon-PR.	SM: Public TS: Kindergarten, elementary, high school, special school P: Family farmers, nutritionists, managers, cooks, teachers (*n* = 125) SD: Environmental, economic, social	Sustainability training; partnerships for environmental preservation, short circuit sales, and certification of organic food; competition and recipe booklet for the use of organic products and valorization of work.
Huston (2018) [69] USA	To highlight how leadership affects the implementation of education forsustainability in two K-6 elementary schools in rural New England.	SM: Public TS: Elementary P: School staff members (*n* = 23) SD: Environmental, economic, social	Education for sustainability; participation in the “Farm-to-School” program; student participation in the local food pantry.
Lagorio et al. (2018) [27] Italy	To use a case study in Italy to illustrate an effective and reliable strategy to reduce food waste in public school canteens.	SM: Public TS: Elementary, high school P: Municipal Councillors of Social Policies and of Education (*n* = 2) SD: Environmental, social	Portion adequacy; food donation.
Lehnerd (2018) [70] USA	To investigate the adoption and the potential impacts of the Farmers’ Market Nutrition Incentive and Farm to School programs.	SM: Not informed TS: Elementary, middle P: Farmer, food service administrators or principals, students (*n* = 721) SD: Environmental, economic, social	Participation in the “Farm to School” program (school garden; local foods)
Powell, Wittman (2018) [71] Canada	To investigate the farm-to-school movement in British Columbia, where concerns related to education and health have been the main vectors of farm-to-school mobilization.	SM: Public TS: Not informed P: Farm-to-school actors (*n* = 30) SD: Environmental, economic, social	Participation in the “Farm-to-School” program (local food, food literacy, school garden).
Roy et al. (2018) [72] India	To explore and further explain the phenomena of supplier participation in addressing the sustainability-oriented objectives of a supply chain.	SM: Public TS: Elementary, upper elementary P: Unit President, purchasing, quality end operation managers (*n* = 4) SD: Environmental, economic, social	Sustainable management of supply chains.
Elkin (2019) [73] USA	To explore the three domains of sustainability of the Farm-to-School program (classroom, cafeteria, and community) developed in a California School District.	SM: Public TS: Elementary, middle P: School district (*n* = 1) SD: Environmental, economic, social	Participation in the “Farm-to-School” program (local food, school garden; teaching about food, farming, and agriculture).
Lopes, Basso, Brum (2019) [74] Brazil	To evaluate the functioning of the market generated by the National School Feeding Program in the school network of Ijuí, RS, Brazil, from the standpoint of short agrifood chains.	SM: Public TS: Elementary P: Education Secretary, nutritionist, school director (*n* = 3) SD: Environmental	School Garden; environmental education.
Santos et al. (2019) [75] Brazil	To implement a school vegetable garden using recyclable materials.	SM: Public TS:Elementary P: School (*n* = 1) SD: Environmental, economic	Organic school garden with recycled material (tires).
Blondin et al. (2020) [25] USA	To assess the Meatless Monday campaign’s nutritional, environmental, and environmental impacts in the National School Lunch Program in a US school district.	SM: Public TS: Not informed P: School district (*n* = 1) SD: Environmental, economic	Reduced meat supply.
Derqui, Grimaldi, Fernandez (2020) [26] Spain	To understand the level of awareness about food waste generated, of interventions applied to minimize it, and to categorize the schools and prioritize a list of interventions to reduce food waste in school canteens.	SM: Public, private TS: Elementary, high school P: School headteachers (*n* = 420) SD: Environmental, economic, social	Certification and training (sustainability); flexible servings; composting; food donation; noise reduction,; communication (adjustment of the quantity produced); reduced use of paper/water/energy.
Izumi et al. (2020) [35] Japan	To explore factors that minimize lunch waste in Tokyo elementary schools and consider how such factors can be modified and applied in US schools.	SM: Public TS: Elementary P: School dietitians (*n* = 5) SD: Environmental, economic	Social norms (avoid waste); exposure to unknown/unappreciated foods; pedagogical practices; portion adequacy; recycling; composting.
Prescott et al. (2020) [76] USA	To identify potential school meal recovery options, their prevalence, and systems factors influencing school food waste recovery across three Northern Colorado school districts.	SM: Public TS: Not informed P: Individuals engaged in food recovery (*n* = 28) SD: Environmental, social	Composting; sharing table; food donation.
Virta, Love (2020) [77] USA	To identify how fishes are implemented in school programs, their impacts, and the enabling factors to support these programs.	SM: Public TS: Elementary, middle, high school P: Seafood processors, Oregon Seafood Commission leaders, school district food service leaders, school kitchen managers (*n* = 6) SD: Environmental, economic, social	Participation in the “Fish to School” program (offer and education about local seafood).
Perez-Neira et al. (2021) [30] Spain	To assess the greenhouse gas emissions reduction of agroecological policies implemented in public food procurement, specifically for school canteens.	SM: Public TS: Pre-School, lementary P: School canteens (*n* = not informed) SD: Environmental, economic, social	Purchase of local, organic, and seasonal food (agro-ecology policies).
Rector et al. (2021) [78] Tanzania	To assess the state of adolescent school nutrition interventions in Dodoma, Tanzania.	SM: Public TS: High School P: School administrators, teachers, students, parents (*n* = not informed) SD: Environmental	School garden.
Toledo (2021) [79] Brazil	To evaluate the “Educational Garden” Program to promote adequate and healthy food in the school environment.	SM: Public TS: Elementary, high school SD: Environmental	School Garden; environmental education.

* Independent schools: Non-profit private schools independent in philosophy, administration, and funding.

**Table 2:** Identified Sustainability recommendations in governmental school food policies documents.

Year (Reference)	Document	Document Type	Responsibility	City/Country	Identified Sustainability Recommendations
Municipality of Barcelona (2020) [82]	This Is Not a Drill. Climate Emergency Declaration, Barcelona.	Declaration	Municipal	Barcelona (Spain)	Implementation and promotion of healthier and low-carbon diets in schools through the use of seasonal, local and organic foods; reduction of animal protein intake (especially red meat) and ultra-processed foods.
The National Food Agency (2021) [84]	Good school meals. Guidelines for primary schools, secondary schools, and youth recreation centers.	Guideline	National	Sweden	Topics on menu planning (including, among others, reducing meat and increasing vegetables, legumes, fruits, and cereals, choosing organic foods, and observing seasonality), measures to prevent food waste, reducing energy consumption, and transport distance.
Brazil (2009)/Brazil (2020) [34,86]	Law *n*° 11.947, from 16 June 2009/Resolution FNDE *n*° 06, May 2020.	Law/Resolution	National	Brazil	Support for sustainable development through purchasing local food from family farming, preference for organic and agroecological food, observation of sustainability in menu planning, and nutrition education actions, seasonality; local traditions.
Italian Ministry of Health (2021) [37]	National guidelines for hospital, care, and school catering Decree 28 October 2021.	Guideline	National	Italy	Seasonality; local, short-chain, organic, and typical foods; environmental protection; animal welfare; local traditions; fair trade; food recovery; reduction of food waste and non-organic residues; food education aimed at conscientious and sustainable consumption; social and environmental criteria for contracts.
Cabinet Office Japan (1954) [88]	School Lunch Program Act.	Law	National	Japan	Respect for nature; a positive attitude towards environmental conservation; a sense of valuing the work of those involved in food production; food education; generation of a correct understanding of the production, distribution, and consumption of food.
United States Department of Agriculture (2015) [89]	Updated Offer versus Serve Guidance for the National School Lunch Program and School Breakfast Program Effective Beginning School Year 2015–2016.	Guidance	National	USA	The possibility of the student refusing some of the foods offered to reduce food waste in school feeding programs.
Santa Catarina (2018) [87]	Law 17.504, 10 April 2018.	Law/Resolution	State	Brazil	Preference for the purchase of organic vegetables by schools, foreseeing a gradual increase in the percentage of purchases.
National Nutrition Council (2017) [81]	Eating and learning together–recommendations for school meals.	Recommendations	National	Finland	Sustainable development and environmental issues concerning food acquisition, food choices, and waste reduction, citing, among others, seasonality; favoring the consumption of domestic vegetables; assembly of dishes by students; possibility of repetition.
Department for Education (2021) [80]	School food standards practical guide.	Guidance	National	England	It recommended sustainable procurement, including the use of fresh, seasonal, sustainable, and locally sourced ingredients, sustainable fish purchase, waste reduction, and school gardens.
Consejo Interterritorial de Sistema Nacional de Salud (2010) [83]	Consensus document about food in educational centers.	Consensus	National	Spain	It informed that the possible incorporation of organic food in school lunches might have advantages about sustainability and protection of the environment. However, it considered no evidence to affirm that organic foods are nutritionally better or safer.
Senate Administration (2017) [85]	Reorganization of the school lunch at open and affiliated all-day primary schools and for support centers in Berlin.	Handout	State	Berlin (Germany)	The establishment of criteria for quality assessment, considered a priority about the price when hiring school food suppliers (organic food corresponds to one of the quality criteria).

**Table 3:** Other available non-governmental school feeding programs/initiatives retrieved from the studies.

Initiative	Country	Description
Food for Life Partnership (FFLP) [94]	England	A program with a whole-school approach that addresses healthy, tasty, and sustainable eating through four areas of development: food quality, food leadership and food culture, food education, and community and partnerships. “Food quality” includes, among other factors, the use of fresh, seasonal, local, and organic foods, meat that meets animal welfare standards, marine conservation certified fish, and eggs from free range hens. “Nutritional education” includes the development of cooking skills, planting food and visiting or receiving visits from farmers, in addition to ethical and environmental issues around food choices.
Eco-Schools [93]	Global	A global program of sustainable schools that aims to train people with an environmental and sustainability conscience. The program is based on seven steps, the: formation of an Eco Committee (in which students play a main role) to discuss environmental and social actions for the school, conduction of a sustainability audit, preparation, monitoring, and evaluation of the action plan, linking of activities to the curriculum, information and involvement with the community, and production of an ecological code that represents the school’s commitment to sustainability.
World Food Programme (WFP)’s Home Grown School Feeding [92]	Global	An initiative in which the World Food Program works with governments to develop school food policies that seek to improve student nutrition and support the local economy through the connection between school food and local farmers.
Smarter Lunchrooms Movement (SLM) [90]	USA	The initiative generated by research in schools is used to create lunchrooms that encourage healthy food choices and reduce waste, using a strategy with little or no cost.
Farm to School (FTS) [91]	USA	It connects schools and local food producers to offer fresh and healthy food to students. It is based on local food purchasing activities, education about food, nutrition, health, agriculture, and hands-on learning activities (school gardens, including visits to local farmers and culinary classes).

## Data Availability

No data availability.

## Supplementary Materials

The following are available online at https://www.mdpi.com/article/10.3390/foods11020176/s1, Table S1: Databases and terms used to search references on sustainability practices adopted in schools; Table S2: Quality criteria of the studies selected for the systematic review.

## Appendix A

**Table A1:** Full-text articles excluded, with reasons.

Author (Year)	Reason for Exclusion
Alexandre et al. (2016) [119]	2
Amarante (2016) [120]	1
Andreatta et al. (2021) [121]	4
Anton-Peset, Fernandez-Zamudio and Pina (2021) [13]	2
Batlle-Bayer et al. (2021) [122]	3
Braun et al. (2018) [123]	4
Brena (2017) [124]	3
Carvalho (2009) [125]	5
Coleman et al. (2011) [126]	1
Colombo et al. (2019) [7]	3
Colombo et al. (2020) [127]	2
Constanty (2014) [128]	4
Constanty and Zonin (2016) [129]	4
Conner et al. (2010) [130]	2
Damapong, Kongnoo and Monarumit (2013) [131]	1
Dirks (2011) [132]	3
Eich (2015) [133]	5
Ellinder et al. (2020) [113]	2
Elnakib et al. (2021) [134]	2
Colombo (2021) [135]	3
Ferderbar (2013) [136]	2
Filippini et al. (2018) [137]	5
Fitzsimmons and O‘Hara (2019) [138]	4
Franzoni (2015) [139]	4
Gaddis and Jeon (2020) [38]	1
Ghattas et al. (2020) [140]	2
Granillo-Maciías (2021) [141]	3
Green (2016) [142]	2
Gregolin et al. (2017) [143]	4
He (2013) [144]	1
Hendler, Ruiz and Oliveira (2021) [145]	3
Henry-Stone (2008) [146]	3
Hodgkinson (2011) [147]	3
Johnston et al. (2009) [148]	5
Jones (2012) [149]	5
Kipfer (2018) [150]	3
Koch (2000) [151]	2
Lalli (2020) [152]	5
Lauffer (2019) [153]	2
Lawless (2013) [154]	3
Lindgren (2020) [112]	3
Løes; Nölting (2011) [28]	1
Løes; Nölting (2009) [155]	1
McCarty (2013) [156]	2
Medina (2009) [157]	5
Melão (2012) [158]	4
Mikkola (2010) [159]	1
Moss Gamblin (2013) [160]	5
Mosiman (2014) [161]	4
Morgan and Morley (2003) [162]	1
Morgan and Sonino (2007) [33]	1
Morgan (2008) [118]	5
Mota, Silva and Pauletto (2021) [163]	5
Muansrichai, Panyasing and Yonvanij (2015) [164]	5
Nunes et al. (2018) [165]	4
Nuutila, Risku-Norja and Arolaakso (2019) [166]	3
Orr (2020) [167]	3
Otsuki (2011) [168]	4
Padilha et al. (2018) [169]	4
Osowski and Fjellström (2018) [170]	1
Polo et al. (2017) [171]	1
Prescott et al. (2019) [172]	2
Rambing et al. (2020) [173]	5
Redman (2013) [174]	2
Resque et al. (2019) [175]	4
Ribeiro, Ceratti and Broch (2013) [176]	4
Rodrigues et al. (2020) [107]	2
Santos et al. (2014) [177]	4
Schachtner-Appel (2019) [178]	2
Scott (2011) [179]	2
Silva and Sousa (2013) [180]	4
Silva and Pedon (2015) [181]	4
Silva, Gehlen and Schultz (2016) [182]	4
Silva, Dias and Amorim (2015) [183]	4
Soares (2011) [184]	4
Soares et al. (2017) [185]	4
Solof (2014) [186]	2
Szinwelski et al. (2015) [187]	4
Trott (2017) [188]	2
Turpin (2009) [189]	4
Vasconcelos, Vieira and Rodrigues (2014) [190]	2
Valadão and Sousa (2018) [191]	1
Wade (2019) [192]	3
Wickramasinghe et al. (2016) [193]	1

Legend—Exclusion criteria: (1) Comments, letters, conferences, reviews, abstracts, reports, undergraduate works, discussion papers, and books, (2) studies carried out outside schools or in which the school was not responsible for the action, (3) studies in which practices were not performed or studies where activities were punctual, (4) studies focused on the supplier or that only reported purchases, and (5) studies that did not describe sustainability practices.
