# Supplementary material for: Sustainability Recommendations and Practices in School Feeding: A Systematic Review
Source: Foods. 2022 Jan 10;11(2):176. doi: 10.3390/foods11020176 (PMC8775006; doi:10.3390/foods11020176)
Supplement: Supplementary file 1 [file foods-11-00176-s001.zip › foods-1523761-supplementary.pdf]

Supplementary file

Table S1. Databases and terms used to search references on sustainability practices adopted in schools.

Database	Search (Jun 30th, 2021)
MEDLINE via Pubmed	("School Feeding"[Title/Abstract] OR "Nutrition Programs and Policies"[Title/Abstract] OR "School Meal"[Title/Abstract] OR "School Meals"[Title/Abstract] OR "School Meal Quality"[Title/Abstract] OR "School Lunch"[Title/Abstract] OR "School Lunches"[Title/Abstract] OR "School Food Service"[Title/Abstract] OR "School Food Services"[Title/Abstract] OR "Brazilian National School Feeding Program"[Title/Abstract] OR "National School Food Program"[Title/Abstract] OR "School Feeding Program"[Title/Abstract] OR "School Feeding Programs"[Title/Abstract] OR "School Feeding Programmes"[Title/Abstract] OR "School Nutrition"[Title/Abstract] OR "School canteens"[Title/Abstract] OR "school canteen"[Title/Abstract]) AND ("Sustainable development"[MeSH Terms] OR "Sustainable development"[Title/Abstract] OR "Waste management"[MeSH Terms] OR "Waste management"[Title/Abstract] OR "Sustainable"[Title/Abstract] OR "Sustainability"[Title/Abstract] OR "Environmental Sustainability"[Title/Abstract] OR "Economic Sustainability"[Title/Abstract] OR "Social Sustainability"[Title/Abstract])
Embase	('school feeding' OR 'nutrition programs and policies' OR 'school meal'/exp OR 'school meal' OR 'school meals' OR 'school meal quality' OR 'school lunch' OR 'school lunches' OR 'school food service' OR 'school food services' OR 'brazilian national school feeding program' OR 'national school feeding program' OR 'school feeding program' OR 'school feeding programs' OR 'school feeding programmes' OR 'school nutrition' OR 'school canteens' OR 'school canteen') AND ('sustainable development'/exp OR 'sustainable development' OR 'waste management'/exp OR 'waste management' OR 'sustainable' OR 'sustainability'/exp OR 'sustainability' OR 'environmental sustainability'/exp OR 'environmental sustainability' OR 'economic sustainability' OR 'social sustainability'/exp OR 'social sustainability')
Web of Science	TS=("School Feeding" OR "Nutrition Programs and Policies" OR "School Meal" OR "School Meals" OR "School Meal Quality" OR "School Lunch" OR "School Lunches" OR "School Food Service" OR "School Food Services" OR "Brazilian National School Feeding Program" OR "National School Food Program" OR "School Feeding Program" OR "School Feeding Programs" OR "School Feeding Programmes" OR "School Nutrition" OR "School canteens" OR "School canteen") AND TS=("Sustainable development" OR "Waste management" OR "Sustainable" OR "Sustainability" OR "Environmental Sustainability" OR "Economic Sustainability" OR "Social Sustainability")
Scopus	(TITLE-ABS-KEY ("school feeding") OR TITLE-ABS-KEY ("nutrition programs and policies") OR TITLE-ABS-KEY ("school meal") OR TITLE-ABS-KEY ("school meals") OR TITLE-ABS-KEY ("school meal quality") OR TITLE-ABS-KEY ("school lunch") OR TITLE-ABS-KEY ("school lunches") OR TITLE-ABS-KEY ("school food service") OR TITLE-ABS-KEY ("school food services") OR TITLE-ABS-KEY ("brazilian national school feeding program") OR TITLE-ABS-KEY ("national school food program") OR TITLE-ABS-KEY ("school feeding program") OR TITLE-ABS-KEY ("school feeding programs") OR TITLE-ABS-KEY ("school feeding programmes") OR TITLE-ABS-KEY ("school nutrition") OR TITLE-ABS-KEY ("school canteens") OR TITLE-ABS-KEY ("school canteen")) AND (TITLE-ABS-KEY ("sustainable development") OR TITLE-ABS-KEY ("waste management") OR TITLE-ABS-KEY ("sustainable") OR TITLE-ABS-KEY ("sustainability") OR TITLE-ABS-KEY ("environmental sustainability") OR TITLE-ABS-KEY ("economic sustainability") OR TITLE-ABS-KEY ("social sustainability"))

Lilacs	(("Alimentação Escolar") OR ("School Feeding") OR ("Programas e Políticas de Nutrição e Alimentação") OR ("Nutrition Programs and Policies") OR ("Merenda Escolar") OR ("School Meal") OR ("School Meals") OR ("School Meal Quality") OR ("School Lunch") OR ("School Lunches") OR ("School Food Service") OR ("School Food Services") OR ("Programa Nacional de Alimentação Escolar") OR ("Brazilian National School Feeding Program") OR ("National School Food Program") OR ("School Feeding Program") OR ("School Feeding Programs") OR ("School Feeding Programmes") OR ("Unidade de Alimentação e Nutrição Escolar") OR ("Unidades de Alimentação e Nutrição Escolares") OR ("School Nutrition") OR ("School canteens") OR ("School canteen")) AND (("Sustainable development") OR ("Desenvolvimento sustentável") OR ("Waste management") OR ("Gerenciamento de resíduos") OR (" Sustentabilidade") OR ("Sustainable") OR ("Sustainability") OR ("Environmental Sustainability") OR ("Sustentabilidade ambiental") OR ("Economic Sustainability") OR ("Sustentabilidade econômica") OR ("Social Sustainability") OR ("Sustentabilidade social")) AND (db:("LILACS"))

Table S2. Quality criteria of the studies selected for the systematic review

Reference	1.Were the practices dentified characterized?	2.Has the practices been implemented in schools?	3. Did the practices present a positive implementation response?	4. Was the study design appropriate?	5. Was the statistical analysis adequate to the objective of the study?	6. Did the results answer the main question?	7. In the case of the schools, was the sample of establishments selected for analysis representative and randomly determined?	Percentage of positive answers (yes) for each study that attained the quality criteria.
Mann (1991)	Y	Y	N	Y	Y	Y	Y	85.71%
Ghiselli (1993)	Y	Y	N	Y	Y	Y	Y	85.71%
Hackes and Shanklin (1999)	Y	Y	N	Y	Y	Y	N	71.42%
Albertse; Mancusi-Materi (2000)	Y	Y	Y	Y	NA	Y	N	85.71%
Wadsworth (2002)	Y	Y	Y	Y	Y	Y	N	85.71%
Lima (2006)	Y	Y	N	Y	NA	Y	N	71.42%
Vogt (2006)	Y	Y	N	Y	Y	Y	N	71.42%
Sonnino (2009)	Y	Y	Y	Y	NA	Y	N	85.71%
Izumi, Alamo and Hamm (2010)	Y	Y	Y	Y	NA	Y	N	85.71%
Baca (2011)	Y	Y	N	Y	Y	Y	N	71.42%
Bennell (2012)	Y	Y	Y	Y	NA	Y	N	85.71%
Bucher (2012)	Y	Y	Y	Y	NA	Y	N	85.71%

Jones et al. (2012)	Y	Y	Y	Y	Y	Y	Y	100%
Lombardini; Lankoski (2013)	Y	Y	N	Y	Y	Y	Y	85.71%
O'Brien (2013)	Y	Y	Y	Y	NA	Y	N	85.71%
Orme et al. (2013)	Y	Y	Y	Y	Y	Y	Y	100%
Rilla (2013)	Y	Y	N	Y	NA	Y	N	71.42%
Shuttleworth (2013)	Y	Y	N	Y	NA	Y	N	71.42%
Barnett (2014)	Y	Y	Y	Y	NA	Y	N	85.71%
Galli et al. (2014)	Y	Y	Y	Y	NA	Y	N	85.71%
He and Mikkelsen (2014)	Y	Y	Y	Y	Y	Y	N	85.71%
Keller (2014)	Y	Y	Y	Y	NA	Y	**N**	85.71%
Bamford (2015)	Y	Y	Y	Y	Y	Y	**N**	85.71%
Black et al. (2015)	Y	Y	N	Y	Y	Y	**N**	71.42%
Coe (2015)	Y	Y	Y	Y	Y	Y	**N**	85.71%
Fabri et al. (2015)	Y	Y	N	Y	Y	Y	**N**	71.42%
Strohl (2015)	Y	Y	Y	Y	NA	Y	**N**	85.71%
Triches (2015)	Y	Y	Y	Y	NA	Y	**N**	85.71%
Fernandes et al.	Y	Y	Y	Y	NA	Y	**Y**	100%

(2016)								
Bareng-Antolin (2017)	Y	Y	Y	Y	Y	Y	**N**	85.71%
Borish, King and Dewey (2017)	Y	Y	Y	Y	Y	Y	**N**	85.71%
Laurie, Faber and Maduna (2017)	Y	Y	Y	Y	Y	Y	**N**	85.71%
Soares et al. (2017)	Y	Y	N	Y	Y	Y	**N**	71.42%
Garcia (2018)	Y	Y	Y	Y	NA	Y	**N**	85.71%
Huston (2018)	Y	Y	Y	Y	NA	Y	N	85.71%
Lagorio et al. (2018)	Y	Y	Y	Y	NA	Y	**N**	85.71%
Lehnerd (2018)	Y	Y	Y	Y	Y	Y	**N**	85.71%
Powell and Wittman (2018)	Y	Y	Y	Y	NA	Y	**N**	85.71%
Roy et al. (2018)	Y	Y	Y	Y	NA	Y	**N**	85.71%
Elkin (2019)	Y	Y	N	Y	Y	Y	**N**	71.42%
Lopes, Basso and Brum (2019)	N	Y	N	Y	NA	Y	**N**	57.14%
Santos et al. (2019)	Y	Y	N	Y	NA	Y	**N**	71.42%
Blondin et al. (2020)	Y	Y	Y	Y	Y	Y	**N**	85.71%
Derqui, Grimaldi and Fernandez (2020)	Y	Y	N	Y	Y	Y	**N**	71.42%
Izumi et al. (2020)	Y	Y	Y	Y	NA	Y	**N**	85.71%

Prescott et al (2020)	Y	Y	N	Y	Y	Y	**Y**	85.71%
Virta; Love (2020)	Y	Y	Y	Y	NA	Y	**N**	85.71%
Perez-Neira et al. (2021)	Y	Y	Y	Y	Y	Y	**N**	85.71%
Rector et al. (2021)	Y	Y	N	Y	NA	Y	**N**	71.42%
Toledo (2021)	Y	Y	Y	Y	NA	Y	**N**	85.71%
